# Lateral Hypothalamus as a Motivation-Cognition Interface in the Control of Feeding Behavior

**DOI:** 10.3389/fnsys.2018.00014

**Published:** 2018-04-16

**Authors:** Gorica D. Petrovich

**Affiliations:** Department of Psychology, Boston College, Chestnut Hill, MA, United States

**Keywords:** lateral hypothalamus, learning, memory, motivation, cognition, feeding, circuitry

## Abstract

Converging evidence for an essential function of the lateral hypothalamus (LHA) in the control of feeding behavior has been accumulating since the classic work conducted almost 80 years ago. The LHA is also important in reward and reinforcement processes and behavioral state control. A unifying function for the LHA across these processes has not been fully established. Nonetheless, it is considered to integrate motivation with behavior. More recent work has demonstrated that the LHA is also required when cognitive processes, such as associative learning and memory control feeding behavior, suggesting it may serve as a motivation-cognition interface. Structurally, the LHA is well positioned within the cerebral hemisphere, with its extensive connectional network across the forebrain-brainstem axis, to link motivational and behavioral systems with cognitive processes. Studies that examined how learned cues control food seeking and consumption have implicated the LHA, but due to methodological limitations could not determine whether it underlies motivation, learning, or the integration of these processes. Furthermore, the identification of specific substrates has been limited by the LHA’s extraordinary complexity and heterogeneity. Recent methodological advancements with chemo-and opto-genetic approaches have enabled unprecedented specificity in interrogations of distinct neurons and their pathways in behaving animals, including manipulations during temporally distinct events. These approaches have revealed novel insights about the LHA structure and function. Recent findings that the GABA LHA neurons control feeding and food-reward learning and memory will be reviewed together with past work within the context of the LHA function as an interface between cognition and motivation.

## Introduction

Learning and motivation are both necessary for the survival of a mammalian organism and their neural substrates have been studied extensively. But these processes have been considered somewhat independently and how they are integrated at a neural circuitry level remains an important area of inquiry (Berridge and Robinson, 2003; Kelley et al., 2005). This mini-review article will outline the evidence that the lateral hypothalamus (lateral hypothalamic area, LHA; Swanson, 2004) functions as an interface between motivation and cognition in the control of feeding behavior. Recent insights gained with advanced methodological approaches will be examined within the context of classic perspectives on the LHA structure and function. The development of opto- and chemo-genetic methods has enabled unprecedented specificity in interrogations of distinct neurons and their pathways. These methods have been used to manipulate the activity of selective, genetically defined neurons within functional circuits during temporally precise events in behaving animals (Fenno et al., 2011; Sternson and Roth, 2014). Novel insights gained with these approaches, particularly that the GABA LHA neurons control feeding and food-reward learning and memory, will be highlighted.

## From A Feeding Center to An Integrative Node within A Distributed Feeding Network

A long history of structural and functional evidence supports a critical role of the LHA in the control of feeding behavior and in reward and reinforcement processes (Hoebel and Teitelbaum, 1962; for reviews see Elmquist et al., 1999; Stuber and Wise, 2016). Early work with electrolytic lesions and electrical stimulations identified the LHA as a “feeding center” in the brain (Hetherington and Ranson, 1940; Anand and Brobeck, 1951) and as one of the areas where reinforcing effects of electrical brain self-stimulations were the most potent (Olds, 1958; for a recent review see Stuber and Wise, 2016). Since then, as our understanding of the organization of the neural substrates underlying motivated behaviors has advanced, the concept of “centers” was replaced with neural networks. The LHA is now considered a core node within a distributed feeding network that integrates different feeding drives and motivates behavior accordingly (Stellar, 1954; Swanson, 2000; De Araujo et al., 2006; Berthoud, 2011; Clifton, 2017; Sternson and Eiselt, 2017).

The LHA is considered to mediate motivational processes underling feeding and other goal-directed behaviors necessary for survival, including translating motivation into action (Mogenson et al., 1980; Mahler et al., 2014; Stuber and Wise, 2016). In addition, the LHA may link motivation and cognition and facilitate their bidirectional influences, as a motivation-cognition interface. Early indication that the LHA can motivate learning comes from classic studies that demonstrated that electrical LHA stimulations enhanced responding for food and mastering a T-maze (Coons et al., 1965; Mendelson and Chorover, 1965). More recent work has shown that the LHA is also needed when cognitive cues drive food and drug reward seeking and consumption (Petrovich et al., 2002; Harris et al., 2005). The LHA is well positioned within the cerebral hemisphere to serve as a motivation-cognition interface. It has extensive connections with the brainstem and hypothalamic areas that process physiological and state signals from the body, as well as with the forebrain cognitive and hedonic systems and areas mediating stress and anxiety (recently reviewed in Reppucci and Petrovich, 2016). Through these connections, the LHA could inform cognitive processes based on organism’s current physiological and behavioral state, and similarly could influence these states based on the outcome of cognitive processes (e.g., learning and memory). Within this framework, new evidence that the LHA is necessary in learning and memory is discussed next.

## The LHA in Reward Learning Acquisition, Memory Recall and Behavioral Expression

There is now strong support that in addition to the ability to influence learning, the LHA is important when learning and memory, in turn, influence the motivation to seek and consume food. In particular, two models of cognitive motivation to eat, cue-induced feeding and conditioned place preference preparations, have implicated the LHA (Petrovich et al., 2002; Harris et al., 2005). These preparations typically have two phases in order to test the influence of food-associated cues (discrete cues or contextual cues) on feeding behavior (food seeking or consumption). The first phase is learning, when cue-food associations are acquired and the second phase is behavioral expression, when the effects of these learned cues on feeding behavior are tested. The behavioral expression involves memory recall of the cue and subsequent induction of food motivation. At which stage during cognitive motivation acquisition and expression the LHA is necessary was not clear until recently. In a study that established the LHA necessity in cue-induced feeding, all manipulations occurred prior to any training and involved unilateral lesions of the LHA (Petrovich et al., 2002). Thus, that work could not determine whether the LHA is solely mediating the *expression* of cue-induced motivation to eat computed elsewhere within the network or whether it is also critical during the *acquisition* of cue-food associations and recall of that memory.

An indication that the LHA may be critically encoding information during the acquisition of reward learning came from a Fos induction imaging study that mapped forebrain recruitment across different stages of Pavlovian cue-food conditioning in rats (Cole et al., 2015a). The LHA was among a few forebrain areas selectively recruited in the learning group that received cue-food pairings compared to controls that received the cue (tone) and food presentations temporally and spatially separated. Thus, the LHA Fos induction patterns were predictive of its critical function during learning; however, until recently, methodological limitations precluded temporally selective manipulations that could establish causality. An exciting, recent study demonstrated that the LHA is necessary for the acquisition and memory storage of cue-food associations. Sharpe et al. (2017) used optogenetic methods in a novel GAD-Cre rat to selectively manipulate GABA neurons within the LHA during cue-food acquisition in a Pavlovian conditioning task. The optogenetic method allowed temporally selective manipulations during the cue (tone) presentations, while leaving food consumption undisturbed. Learning and memory was assessed by the cue’s ability to drive food-seeking behavior (approach to the food receptacle). They showed that optogenetic inhibition of the LHA^GABA^ neurons selectively during cue presentations disrupted the *acquisition* and *memory* of cue-food association.

These findings are consistent with other recent evidence that the LHA^GABA^ neurons are critical in the control of feeding behavior (Jennings et al., 2013; Nieh et al., 2015; O’Connor et al., 2015) and together suggest that a common substrate may mediate food learning and consumption. However, the LHA^GABA^ neurons are not homogenous and distinct subpopulations appear to mediate different aspects of food motivated behaviors (see “GABA Neurons**”** section). Additionally, a recent study found that some ORX neurons express GAD1 (Mickelsen et al., 2017), which is important for the interpretation of the above manipulations of GAD-neurons (Sharpe et al., 2017), given the ORX function in learning and motivation (see “ORX Neurons” section). Thus, identifying which subpopulation of the LHA neurons support learning and how they interact with other local neurons is imperative to our understanding of the LHA function. Equally important is identifying which specific inputs control the critical LHA neurons during reward learning and memory and which outputs mediate behavioral control.

## The LHA Contains Heterogeneous Regions and Neural Substrates

The LHA is a large and complex structure and its different regions have distinct functions (Khan, 2013). There are also interspecies differences (e.g., Bittencourt et al., 1998; Chometton et al., 2014, 2016), and therefore the evidence from rodent studies discussed here should be interpreted with caution in regard to the LHA in primates and other species. Classic studies have shown that glutamate receptor agonists and neuropeptide Y (NPY) infusions into the perifornical area of the LHA stimulate feeding (Flood and Morley, 1991; Stanley et al., 1993, 2011), and at least the NPY effects are mediated via enhanced motivation to eat (Flood and Morley, 1991). That area contains neurons that express orexigenic neuropeptides, orexin/hypocretin (ORX) and melanin concentrating hormone (MCH), which when released centrally stimulate feeding behavior (Nahon et al., 1989; Qu et al., 1996; Broberger et al., 1998; Sakurai et al., 1998; de Lecea et al., 1998; Swanson et al., 2005). ORX is also critical for arousal and wakefulness, which is necessary for all goal-directed behaviors (Boutrel et al., 2010; Scammell et al., 2017). An overlapping area is also where reinforcing effects of electrical stimulations (brain stimulation reward) are sensitive to food deprivation and adipose-produced hormone leptin (Abrahamsen et al., 1984; Fulton et al., 2000). Thus, the effects of glutamate receptor agonists and NPY infusions likely mimic naturally occurring release when inputs relaying physiological or cognitive signals drive feeding behavior. Connectional data discussed next support this premise.

The perifornical region, including the suprafornical area for which detailed connections were recently established (Hahn and Swanson, 2010) could receive information regarding the energy balance and short-term, hunger and satiety signals, via inputs from the arcuate nucleus of the hypothalamus (ARH), including NPY neurons. It also receives inputs from other hypothalamic and brainstem areas, including behavioral state systems (e.g., dorsal raphe; Nectow et al., 2017), as well as inputs from the striatum (e.g., nucleus accumbens shell; O’Connor et al., 2015) and pallidum, particularly the bed nuclei of the stria terminalis (BST) and substania innominata (Dong and Swanson, 2004, 2006a,b; Hahn and Swanson, 2010). The ARH NPY neurons also express agouti-related peptide (AgRP) and GABA, which, interestingly, are differently controlling rapid (NPY and GABA) vs. prolonged (AgRP) feeding (Krashes et al., 2013). The perifornical region also receives inputs from cortical areas processing cognitive information, including the medial prefrontal cortex, the basolateral area of the amygdala (BLA) and the hippocampal formation (all presumed to be glutamatergic) and from the paraventricular thalamus (PVT), which is interconnected with these areas (Hahn and Swanson, 2010; Mena et al., 2013; Hsu et al., 2015; Kanoski and Grill, 2015; Sun et al., 2015; Reppucci and Petrovich, 2016). A third type of inputs to this area is from the regions well known for their role in stress and anxiety, the central nucleus of the amygdala (CEA) and BST, and their projecting neurons are GABAergic (Swanson and Petrovich, 1998; Dong et al., 2001; Hahn and Swanson, 2010; Kim et al., 2013; Reppucci and Petrovich, 2016). These parts of the CEA and BST, along with the LHA, are interconnected with the parabrachial nucleus (PB), including the areas that relay pain information (Bernard et al., 1993; Alden et al., 1994; Bester et al., 1997). This is notable, as the CEA and PB have been established in suppression of feeding (Petrovich et al., 2009; Carter et al., 2013; Cai et al., 2014).

How the LHA inputs are integrated and how local neurons are organized to compute specific outputs are fundamental questions that classic methods could not address, because heterogeneous LHA populations of neurons are intermingled. In addition to the ORX and MCH populations, the LHA contains GABAergic and glutamatergic neurons and distinct combinations of various receptors (e.g., leptin; Leinninger, 2011), peptides, and opioids (reviewed in Bonnavion et al., 2016). The burgeoning opto- and chemo-genetic methods, which enable cell-specific interrogations, are beginning to answer these questions, but they are also revealing additional complexity in the LHA organization.

### GABA Neurons

Cell-specific manipulations have identified the LHA^GABA^ neurons as a critical substrate in the control of feeding behavior and reward associative learning and memory, as discussed above. Consistent with that notion, activation of the LHA^GABA^ neurons stimulated (Jennings et al., 2013), while their inhibition halted (O’Connor et al., 2015) feeding. However, relevant to the interpretation of these results regarding the LHA^GABA^ physiological function, chemogenetic manipulations found that they mediate non-specific consummatory behaviors directed at food and non-food items (Navarro et al., 2016). Furthermore, the LHA^GABA^ neurons are diverse and do not function in isolation (e.g., Jennings et al., 2015; Mickelsen et al., 2017; Qualls-Creekmore et al., 2017). Additionally, whether they are distributed or localized within a specific area (e.g., perifornical) is not addressed in these studies. Distinct subpopulations of LHA^GABA^ neurons (Vgat-expressing neurons that contain neither MCH or ORX) are believed to mediate food motivation (measured by approach behavior) vs. consumption, based on their distinct activation patterns (Jennings et al., 2015). Furthermore, selective manipulations of the Galanin-expressing subpopulation (GABA^GAL^) indicated that they mediate food seeking behavior (operant responding for sucrose reward) but not the chow consumption or compulsive locomotion that were observed after stimulation of total LHA^GABA^ neurons (Qualls-Creekmore et al., 2017).

There is also strong indication that different outputs from the LHA^GABA^ neurons mediate different aspects of feeding behavior. Activation of a subset of these neurons that project to the paraventricular nucleus of the hypothalamus stimulated feeding (Wu et al., 2015), while activation of the projections to the ventral tegmental area (VTA) produced more complex results (reviewed in this research topic (Tyree and de Lecea, 2017). Activation of the LHA^GABA^–VTA neurons increased feeding duration and produced aberrant licking and gnawing behaviors (Nieh et al., 2015). Notably, the LHA GABA^GAL^ neurons do not send direct projections to the VTA, the pathway hypothesized to mediate compulsive locomotion (Qualls-Creekmore et al., 2017). Instead, they innervate ORX neurons (Laque et al., 2015), which are important in behavioral state control and motivation (see “ORX Neurons” section). The results of manipulations of GAD-Cre rat LHA neurons projecting to VTA indicate that pathway conveys information about reward predictions for learning but not for behavioral control (Sharpe et al., 2017). Additional complexity was revealed recently, in that the LHA^GABA^–VTA pathways can induce feeding or rewarding effects depending on the frequencies of their stimulation (Barbano et al., 2016). Interestingly, it was hypothesized that different behavioral outcomes were mediated by different neurotransmitters released with low and high stimulation frequencies from the same fibers (Barbano et al., 2016).

### Glutamatergic Neurons

The glutamatergic (VGlut2-expressing) LHA neurons are critical in feeding (Stamatakis et al., 2016). The BST inhibitory inputs were demonstrated to innervate and suppress these neurons, which initiated consumption (Jennings et al., 2013). Similar to the LHA^GABA^, glutamatergic LHA neurons are diverse. Notably, most ORX neurons are glutamatergic, while subpopulations of MCH neurons are glutamatergic or GABAergic (reviewed in Bonnavion et al., 2016). Thus, determining how different subpopulations of glutamatergic and GABA LHA neurons are integrated and which local and external inputs control them are important areas of future inquiries. In that regard, anatomical evidence suggests that in addition to the BST, converging inputs from the CEA could provide inhibition of the LHA glutamatergic neurons, including ORX neurons (Swanson and Petrovich, 1998; Reppucci and Petrovich, 2016; also the CEA-CRH neurons can activate ORX neurons; Winsky-Sommerer et al., 2004).

### ORX Neurons

The ORX neurons are important for feeding as well as behavioral state control and motivation (de Lecea et al., 1998; Boutrel et al., 2010; Hurley and Johnson, 2014; Mahler et al., 2014; Sakurai, 2014), including arousal associated with changes in energy balance and feeding (Yamanaka et al., 2003; González et al., 2016). Relevant to the proposed LHA function as a motivation-cognition interface, ORX neurons are critical during reward associative learning as well as when learned food cues motivate feeding behavior. It is now well established that ORX neurons are activated in response to discrete and contextual food cues (Harris et al., 2005; Choi et al., 2010; Petrovich et al., 2012; Hassani et al., 2016) and their signaling via type 1 receptor (ORX-R1) mediates cue-induced feeding and operant responding for food (Nair et al., 2008; Borgland et al., 2009; Sharf et al., 2010; Cason and Aston-Jones, 2013; Cole et al., 2015b). They are also recruited during cue-food associative learning (Cole et al., 2015a) and ORX-R1 signaling modulates the acquisition and extinction of cue-food associations (Keefer et al., 2016). Thus, determining how external and local inputs control ORX neurons and how their outputs to different targets (Ho and Berridge, 2013), along with those from other LHA neurons, sum up to coordinate behavior is a pressing area of interest.

### MCH Neurons

In addition to its essential function in the homeostatic regulation of food consumption and body weight the MCH is important in reward learning and memory (for reviews see Adamantidis and de Lecea, 2009 and in this topic Diniz and Bittencourt, 2017). The MCH regulates reward-seeking behavior and MCH-Receptor1 signaling is necessary for cue-induced feeding (Sherwood et al., 2015; Sita et al., 2016). Given the widespread distribution of its fibers and receptors, the MCH could affect multiple learning and memory systems (Diniz and Bittencourt, 2017). Notably, anti-MCH infusions in the hippocampal formation affected the latency to seek food in a working-memory spatial task, demonstrating a role in guiding learned behavioral responses (Sita et al., 2016).

## Integrative Processing Across LHA Circuitry

The integration of food and energy sensory information may occur at multiple sites within the LHA circuitry. The ARH neurons, which are considered to relay energy-related sensory signals, appear to already integrate that information and respond in an anticipatory way (Chen et al., 2015) reviewed in Seeley and Berridge (2015). The activity of orexigenic, AgRP (NPY/GABA) neurons was high in fasted mice, as expected, but it decreased as soon as food was presented and eating began. The opposite was found for the anorexigenic, POMC/CART neurons. If food was removed during a meal, these patterns were reset and AgRP neurons increased activity, while the POMC/CART neurons decreased activity. These patterns suggest that the activity of the ARH neurons is regulated in anticipation of energy gain from a meal and that the incoming sensory information is continuously updated. The ARH and LHA bi-directionally communicate, and thus the LHA could update ARH neurons and guide their responding, in accordance with its proposed role in motivation-cognition integration.

Indeed, it is very likely that incoming sensory and processed cognitive information is continuously updated within the LHA circuitry. In that regard, the LHA may also contribute to the recently revealed circuitry underlying motivational state (hunger/satiety) control over insular cortex (AI) processing during responding to food cues (Livneh et al., 2017). The LHA is well positioned to communicate across that system, as it is connected with each component of the AgRP-PVT-BLA-AI circuitry.

## Concluding Remarks

Recent technologies, which enabled selective interrogations of specific neurons and their circuitries, have greatly advanced the field. These methods have revealed a novel LHA function in learning and memory and identified cell-specific substrates and their pathways in the control of feeding behavior. They are also revealing another dimension of LHA structural complexity. Future work will require thoughtful synergies across genetic, anatomical, and behavioral approaches to unearth the organization of the LHA structure and how it functions to control feeding and other motivated behaviors.

## Author Contributions

The author confirms being the sole contributor of this work and approved it for publication.

## Conflict of Interest Statement

The author declares that the research was conducted in the absence of any commercial or financial relationships that could be construed as a potential conflict of interest.
